# Investigation of the Effect of Substituents on Electronic and Charge Transport Properties of Benzothiazole Derivatives

**DOI:** 10.3390/molecules27248672

**Published:** 2022-12-08

**Authors:** Ahmad Irfan, Abul Kalam, Abdullah G. Al-Sehemi, Mrigendra Dubey

**Affiliations:** 1 Department of Chemistry, College of Science, King Khalid University, P.O. Box 9004, Abha 61413, Saudi Arabia; 2 Research Center for Advanced Materials Science, College of Science, King Khalid University, P.O. Box 9004, Abha 61413, Saudi Arabia; 3 Soft Materials Research Laboratory, Discipline of Metallurgy Engineering and Materials Science, Indian Institute of Technology Indore, Indore 453552, India

**Keywords:** organic semiconductor materials, benzothiazole, density functional theory, optical properties, charge transport

## Abstract

A series of new benzothiazole-derived donor–acceptor-based compounds (**Comp1–4**) were synthesized and characterized with the objective of tuning their multifunctional properties, i.e., charge transport, electronic, and optical. All the proposed structural formulations (**Comp1–4**) were commensurate using FTIR, ^1^H NMR, ^13^C NMR, ESI-mass, UV–vis, and elemental analysis techniques. The effects of the electron-donating group (-CH_3_) and electron-withdrawing group (-NO_2_) on the optoelectronic and charge transfer properties were studied. The substituent effect on absorption was calculated at the TD-B3LYP/6-31+G** level in the gas and solvent phases. The effect of solvent polarity on the absorption spectra using various polar and nonpolar solvents, i.e., ethanol, acetone, DMF, and DMSO was investigated. Light was shed on the charge transport in benzothiazole compounds by calculating electron affinity, ionization potential, and reorganization energies. Furthermore, the synthesized compounds were used to prepare thin films on the FTO substrate to evaluate the charge carrier mobility and other related device parameters with the help of I-V characteristic measurements.

## 1. Introduction

Organic semiconductors have become indispensable because of their cheap, malleable, lightweight, and environmentally friendly properties [1,2,3,4,5,6,7,8]. Advances in organic semiconductor materials (OSMs) in organic field effect transistors (OFETs), photovoltaics, and organic thin film transistors (OTFTs) have motivated scientists to explore materials [9,10,11,12,13,14,15,16,17,18,19,20,21]. The versatility of OSMs allows many interesting properties to be tuned because organic materials such as benzothiazole have promising applications in fluorescence sensors, OLEDs, solar cells, OFETs, OTFTs, etc., and have attracted great interest [22,23,24,25,26].

**Figure 1:**
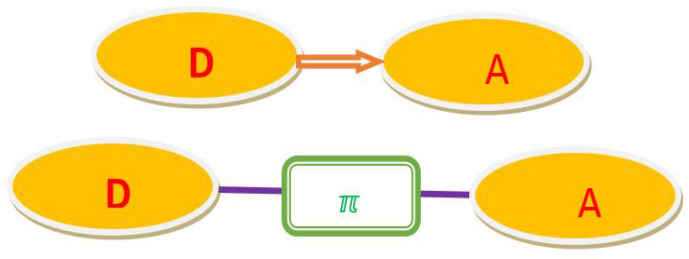
Donor–acceptor (DA) model system diagram.

Low band gap materials have attracted the attention of the scientific community because of their high tendency to harvest photons of visible wavelength, redox-tunable quality, and use in ambipolar transistors [27,28,29,30,31,32,33]. Essentially, a promising strategy is to develop molecules that carry electron-donating moiety (D) and electron-acceptor moiety (A) that can match the charge carrier injection and receive both holes and electrons. The most important materials to date have been modifications of the HOMO/LUMO (highest/lowest occupied/unoccupied molecular orbital) of the molecule, which can be obtained by the simple introduction of the suitable D and A units in the molecule [34,35,36]. In such compounds, the interaction of the HOMO, which donates the electron (D), and the LUMO, which accepts the electron (A), results in a narrowing of the gap [37]. At present, HOMO and LUMO molecule levels can be controlled, making those systems more attractive for use in OSMs [38]. The benzothiazole group has been described as a valuable receptor. It provides a D-π-A compound that exhibits two-photon absorption (TPA) compared with asymmetrical chromophores [39]. The donor–acceptor (DA) model system diagram is illustrated in Figure 1. By adopting the benzothiazole unit as a well-made acceptor, the changes made by various donors can further increase the TPA.

The computational probing of the electronic structure of compounds has made a significant contribution to estimating known and unpredicted properties. In this context and to guide the integration of smaller energy gap materials, computational methods have been widely used to predict energy gaps [40,41,42]. Computational chemistry is accompanied by a variety of applications in fields such as materials science, energy, engineering, nanoscience, etc. [43]. The density functional theory (DFT) seems to be a good choice and has been established as an effective means of investigating the electronic, charge transfer, and physical properties associated with various materials. The DFT method provides the most accurate results for electronic, optical, and charge transport properties that are in the closest agreement with experimental evidence [44,45,46]. In addition, computational calculations are extensively used to determine properties and provide spectral data. There are many studies on the calculation using DFT of the various properties of certain thiophene and benzothiazoles, such as ultraviolet–visible spectroscopy (UV–vis), HOMO-LUMO energy, the molecular electrostatic potential, and charge distribution [47,48].

**Figure 2:**
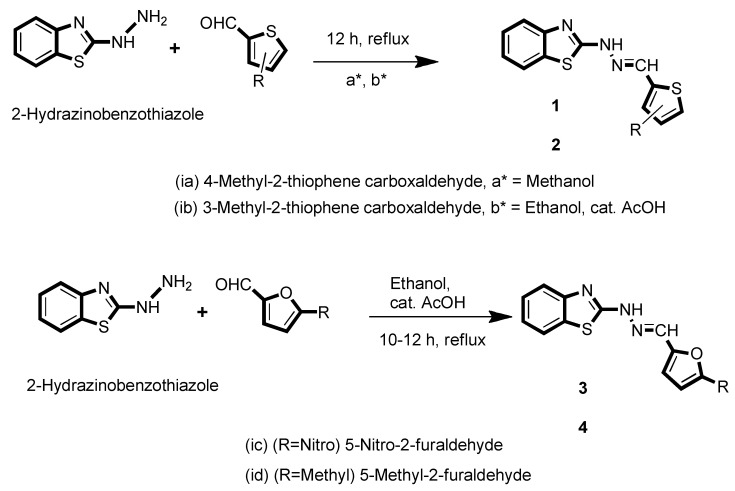
The structures of benzothiazole derivatives.

We synthesized various dyes aiming to investigate their OFET properties. First-principle methods were used to discover the impact of electron-donating -CH_3_ and -NO_2_ groups on the charge transport and optoelectronic properties. We anticipated that the substitution of positions 3 and 4 for electron-donating groups (EDGs) and position 5 for the electron-withdrawing group (-NO_2_) would boost properties of interest (Figure 2).

## 2. Methods

### 2.1. Computational Details

The optimization of ground state (S_0_) geometries of benzothiazole compounds was achieved by density functional theory (DFT) [49,50,51,52] at the B3LYP/6-31+G** level. The frequency calculations were performed at the B3LYP/6-31+G** level. There was no negative frequency obtained, which indicated that the optimized structures were suitable for further studies. Earlier studies demonstrated that B3LYP was able to calculate charge transport [53]. Here, electron affinity (EA), ionization potential (IP), and reorganization energy for hole/electron (λ_hole_/λ_elec_) values were computed at the B3LYP/6-31G** level. The optimization of geometries in an excited state (S_1_) was achieved by time-dependent DFT [54] at the TD-B3LYP/6-31+G** level. The absorption (λ_max_) was assessed at the TD-B3LYP/6-31+G** level in gas-phase and solvents (ethanol, acetone, DMF, and DMSO) with the Gaussian16 package [55] using self-consistent reaction field (SCRF) theory with a polarized continuum model (PCM) [56].

### 2.2. Experimental Details

All the characterization spectra of FTIR, ^1^HNMR, ^13^C NMR, and ESI-mass were incorporated in supporting files as Figures S1–S17.

#### 2.2.1. Synthesis of **Comp1**

Methanolic solution of 4-methyl-2-thiophenecarboxaldehyde (0.332 g, 2 mmol) was added dropwise to the solution of 2-hydrazino benzothiazole (0.252 g, 2 mmol) dissolved in methanol. The resulting mixture was continuously stirred for 12 h under reflux conditions. The resulting pale yellow-colored precipitate of **Comp1** was further filtered and washed with methanol and ether thrice (yield: 0.428 g, 78%).

^1^H NMR (500 MHz, DMSO, ppm): 2.21 (s, 3H, -CH_3_), 7.09 (s, 1H, thiophene-H), 7.19 (s, 1H, thiophene-H), 7.22 (s, 2H, Ar-H), 7.28 (s, 1H, Ar-H), 7.73 (s, 1H, Ar-H), 8.26 (s, 1H, =CH), 12.15 (s, 1H, -NH). ^13^C NMR (500 MHz, DMSO, ppm): 15.74, 121.98, 122.14, 123.79, 126.47, 132.23, 138.19, 144.10, 167.04. FTIR (ATR, cm^−1^): 1615 (C=N), 1575 (C=N; benzothiazole ring). UV–vis spectrum [(0.5 × 10^−5^ M; lmax, solvent), (e; L M^−1^ cm^−1^)]: 353 (acetone; 59,000), 360 (DMF; 59,000), 362 (DMSO; 58,500). Anal. calcd. for C_13_H_11_N_3_S_2_: C, 57.12; H, 4.06; N, 15.37; S, 23.46. Found C, 57.16; H, 4.10; N, 15.41; S, 23.42. HR-MS [M+H]^+^: 274 (calcd. 274).

#### 2.2.2. Synthesis of **Comp2**

Ethanolic solution of 3-methyl-2-thiophenecarboxaldehyde (0.229 g, 1.815 mmol) was added dropwise to the solution of 2-hydrazino benzothiazole (0.300 g, 1.815 mmol) dissolved in ethanol in the presence of 5 drops of acetic acid. The resulting mixture was continuously stirred for 12 h under reflux conditions. The resulting off-white-colored precipitate of **Comp2** was further filtered and washed with ethanol and ether (yield: 0.372 g, 75%).

^1^H NMR (500 MHz, DMSO, ppm): 2.38 (s, 3H, -CH_3_), 7.01 (s, 1H, Fural-H), 7.14 (s, 1H, Fural-H), 7.34 (s, 1H, Ar-H), 7.43 (s, 1H, Ar-H), 7.57 (s, 1H, Ar-H), 7.79 (s, 1H, Ar-H), 8.40 (s, 1H, =CH), 12.12 (s, 1H, -NH). FTIR (ATR, cm^−1^): 1616 (C=N), 1576 (C=N; benzothiazole ring). ^13^C NMR (500 MHz, DMSO, ppm): 14.52, 121.01, 121.24, 122.70, 124.52, 128.81, 131.31, 143.01, 167.01. UV–vis spectrum [(0.5 × 10^−5^ M; l_max_, solvent), (*ε*; L M^−1^ cm^−1^)]: 352 (ethanol; 55,150), 353 (acetone; 55,150), 358 (DMF; 55,000), 361 (DMSO; 55,050). Anal. calcd. for C_13_H_11_N_3_S_2_: C, 57.12; H, 4.06; N, 15.37; S, 23.46. Found C, 57.11; H, 4.09; N, 15.41; S, 23.40. HR-MS [M+H]^+^: 274 (calcd. 274).

#### 2.2.3. Crystal Structure of **Comp2**

**Figure 3:**
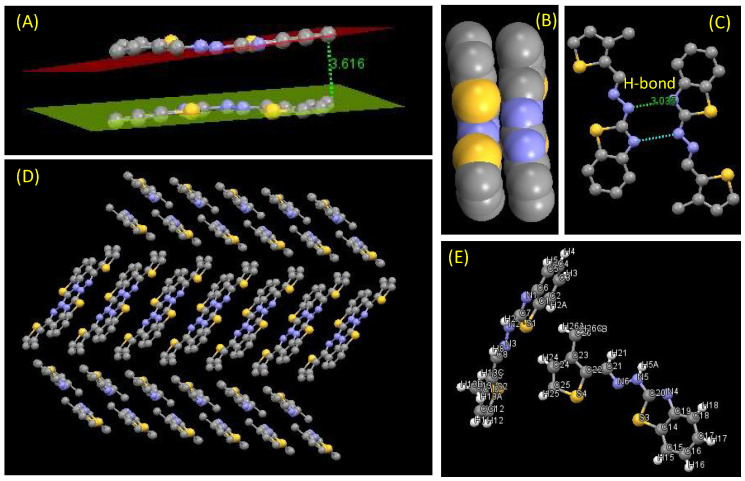
(**A**) Two adjacent molecules showing the π-π stacking interaction also displayed with the help of (**B**) the space fill model, (**C**) H-bonding interaction between two laterally close molecules (bond length shown in Å), (**D**) extended network of molecules though weak interactions (π-π stacking and H-bonding), and (**E**) labeling of each atom shown in ball stick model.

**Figure 4:**
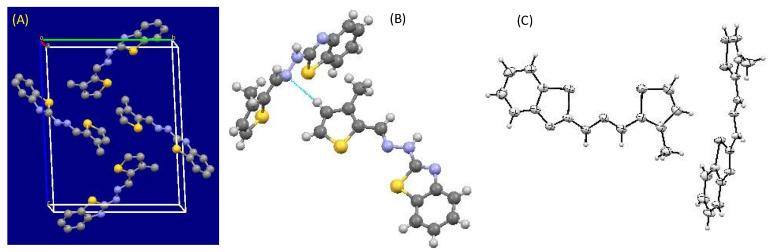
(**A**) View along the a* axis displaying the packing of molecules in monoclinic fashion, (**B**) Van der Waal interaction present between two molecules, and (**C**) ORTEP diagram of **Comp2** (ellipsoids at 40% probability).

Empirical formula: C_13_H_11_N_3_S_2_; formula weight: 273.37; crystal system: triclinic; space group P-1, unit cell dimensions a = 5.8300 (12) Å, α = 87.37 (3)°, b = 13.365 (3) Å, β = 82.25 (3)°, c = 16.684 (3) Å and γ = 83.70 (3)°; volume 1279.7 (5) Å^3^; Z = 4; density (calculated) 1.419 Mg/m^3^ (see Figure 3 and Figure 4).

#### 2.2.4. Synthesis of **Comp3**

Ethanolic solution of 5-nitro-2-furaldehyde (0.256 g, 1.815 mmol) was added dropwise to the solution of 2-hydrazino benzothiazole (0.300 g, 1.815 mmol) dissolved in ethanol in the presence of 5-7 drops of acetic acid. The resulting mixture was continuously stirred for 10 h under reflux conditions. The resulting orange-colored precipitate of **Comp3** was further filtered and washed with ethanol and ether (yield: 0.377 g, 72%).

^1^H NMR (500 MHz, DMSO, ppm): 7.16 (d, 2H, Ar-H), 7.33 (d, 1H, Ar-H), 7.45 (s, 1H, Ar-H), 7.79 (d, 2H, Fural-H), 8.10 (s, 1H, =CH), 12.71 (s, 1H, -NH). ^13^C NMR (500 MHz, DMSO, ppm): 108.99, 115.01, 121.91, 122.04, 126.42, 133.41, 148.35, 154.68, 156.52, 167.08. FTIR (ATR, cm^−1^): 1622 (C=N), 1581 (C=N, benzothiazole). UV–vis spectrum [(0.5 × 10^−5^ M; λ_max_, solvent), (*ε*; LM^−1^ cm^−1^)]: 430 (DMF; 57,500), 445 (DMSO; 57,400). Anal. calcd. for C_13_H_11_N_3_S_2_: C, 50.00; H, 2.80; N, 19.43; S, 11.12. Found C, 50.11; H, 2.83; N, 19.40; S, 11.15. HR-MS [M+Na]^+^: 311 (calcd. 311). [M+H]^+^; 289 (289).

#### 2.2.5. Synthesis of **Comp4**

A solution mixture of 2-hydrazino benzothiazole (0.300 g, 1.815 mmol) with 5 drops of acetic acid was separately prepared and allowed to react with 5-methylfurfrualdehyde (0.200 g, 1.815 mmol). The resulting solution mixture was refluxed for 12 h to further obtain the light brown precipitate of compound **Comp4,** which was washed with ethanol and ether (yield: 0.355 g, 76%).

^1^H NMR (500 MHz, DMSO, ppm): 2.36 (s, 3H, -CH_3_), 6.26 (s, 1H, Fural-H), 6.74 (s, 1H, Fural-H), 7.08 (s, 1H, Ar-H), 7.30 (s, 1H, Ar-H), 7.38 (s, 1H, Ar-H), 7.74 (s, 1H, Ar-H), 7.93 (s, 1H, =CH), 12.11 (s, 1H, -NH). ^13^C NMR (500 MHz, DMSO, ppm): 14.17, 117.06, 122.00, 122.13, 131.52, 132.94, 139.85, 149.14, 152.20, 166.99. FTIR (ATR, cm^−1^): 1622 (C=N), 1607 (C=N, benzothiazole). UV–vis spectrum [(0.5 × 10^−5^ M; λ_max_, solvent), (*ε*; L M^−1^ cm^−1^)]: 343 (acetone; 59,000), 344 (ethanol; 59,000), 350 (DMF; 59,000), 353 (DMSO; 58,950). Anal. calcd. for C_13_H_11_N_3_OS: C, 60.68; H, 4.31; N, 16.33; S, 12.46. Found C, 60.26; H, 4.40; N, 16.30; S, 12.39. HR-MS [M+H]^+^: 258 (calcd. 258), *[M+Na]^+^*; 280 (calcd. 280).

## 3. Results and Discussion

### 3.1. Electronic Properties

The E_HOMO_, E_LUMO_, and E_gap_ of S_0_ and S_1_ of the benzothiazole compounds are provided in Table 1. The trend in the S_0_ E_HOMO_ was: **Comp4** (−5.52) > **Comp2** (−5.58) > **Comp1** (−5.59) > **Comp3** (−6.18). The trend in E_LUMO_ at S_0_ was: **Comp2** (−1.88) > **Comp4** (−1.92) > **Comp1** (−1.95) > **Comp3** (−3.35). The effect of -NO_2_ in place of -CH_3_ in the furan-based derivative was more useful for tuning the energy values of frontier molecular orbitals (FMOs), i.e., **Comp3**. One can see that the substitution of -NO_2_ group lowered the E_HOMO_ and E_LUMO_ values of **Comp3,** resulting in reducing the E_gap_, i.e., 2.83 eV at S_0_.

**Figure 5:**
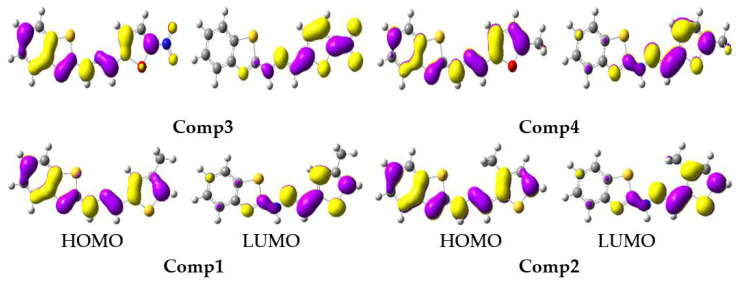
The frontier molecular orbitals distribution of benzothiazoles at a ground state (contour value = 0.035).

**Figure 6:**
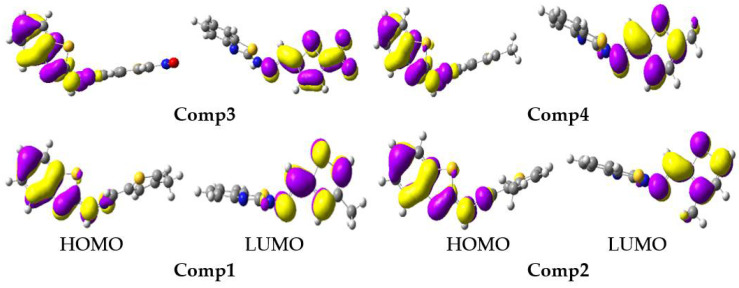
The frontier molecular orbitals distribution of benzothiazoles in an excited state (contour value = 0.035).

The tendency in the S_1_ E_HOMO_ was: **Comp4** (−4.73) > **Comp2** (−4.76) > **Comp1** (−4.77) > **Comp3** (−5.13), while in the S_1_ E_LUMO_ it was: **Comp2** (−2.55) > **Comp4** (−2.56) > **Comp1** (−2.61) > **Comp3** (−4.01). The substitution of -NO_2_ at position-5 on the furan ring led to lowering the energy levels of the HOMO and LUMO in **Comp3**, which reduced the E_gap_ of S_1_, i.e., 1.12 eV, and was advantageous for charge transport and optoelectronic properties. The FMOs at S_0_ and S_1_ are illustrated in Figure 5 and Figure 6, respectively. The HOMO was delocalized on entire molecules in all studied systems, and the LUMO was localized toward the thiophene/furan moiety in **Comp1**, **Comp2,** and **Comp4**, while -NO_2_ in **Comp3** was also involved in the formation of the LUMO showing an intra-molecular charge transfer (ICT) from H→L. At S_1_, the HOMO was delocalized on the benzothiazoles ring in all the derivatives, while the charge density at the LUMO was nearly the same as S_0_.

The work functions (*ϕ*) of Ag (Al) were 4.74 (4.08 eV) [57]. The hole/electron injection energy barricades (HIE/EIE) of benzothiazoles to the Al electrode were computed as (*ϕ*-E_HOMO_) and (E_LUMO_-*ϕ*), correspondingly. The EIE from **Comp1**–**Comp4** to Al were 2.13, 2.20, 0.73, and 2.16 eV, respectively. The HIE for **Comp1**–**Comp4** were 1.51, 1.50, 2.00, and 1.44 eV, correspondingly. The calculated EIE from **Comp1**–**Comp4** to Ag were 2.79, 2.86, 1.39, and 1.82 eV, respectively. The HIE for **Comp1**–**Comp4** were 0.85, 0.84, 1.44, and 0.78 eV, respectively. These outcomes suggested that the substitution of the -NO_2_ group at position 5 on the furan moiety in **Comp3** would encourage the development of the electron injection, while the -CH_3_ group might top up the hole injection aptitude, see Table 2.

### 3.2. Absorption Spectra

Absorption (λ_max_), oscillator strengths (*f*), and the percentage contribution of the transitions of the FMOs to the studied derivatives of benzothiazole on the TD-B3LYP/6-31+G** level in gas-phase and various solvents are shown in Table 3. The solvent effect has attracted much attention because of many of the chemical processes that occur in the solution phase. In an effort to explore the effect of the solvent on the quantum–chemical calculations of the absorption maxima, the gas phase of the studies consisted of molecules (as a control), and then the data were compared with the λ_max_ computed in a solvent such as ethanol, acetone, DMF, and DMSO. The effects of the substituents on the λ_max_ at the TD-B3LYP/6-31+G** level in the gas phase and the solvent stage were compared and discussed. The effect of solvent polarity on the absorption spectra was measured in polar and non-polar solvents (ethanol, acetone, DMF, and DMSO), as demonstrated in Figures S5 and S6, and corresponding data are tabulated in Table S1.

Here, the first λ_max_ band was noticed at 358 nm related to H→L (S_0_→S_1_), whereas the second was from H→L + 1 at 281 nm for **Comp1** in the gas phase. The first λ_max_ band was observed at 363 nm corresponding to H→ L (S_0_ → S_1_), while the second was from H→L + 1 at 281 nm for **Comp1** in ethanol. Additionally, no significant effect was observed on λ_max_ from the gas phase to solvent in **Comp1**, **Comp2,** and **Comp4**. The solvent polarity also had no significant effect on λ_max_. In the gas phase, the substitution of -NO_2_ at position 5 of furan in **Comp3** led to a red shift, i.e., 93 nm in the first band and 42 nm in the second band compared with **Comp1**. We noted that a significant effect was observed in λ_max_ from the gas phase to solvent in **Comp3**, i.e., 481 nm in ethanol vs. 451 nm in the gas phase. The computed data were in good agreement with the experimental findings, see Table S1. The substitution of -NO_2_ at position 5 of furan in **Comp3** led to a redshift in λ_max_ from the gas phase to ethanol, acetone, DMF, and DMSO, i.e., 30, 30, 33, and 33 nm for the first band and 12, 12 13, and 13 nm for the second. The effect of electron acceptor and electron donor moiety was mainly on the first λ_max_ band, i.e., the introduction of -NO_2_ at position 5 of furan in **Comp3** tuned the wavelength toward a longer wavelength.

All electronic transitions and corresponding oscillator strengths in the absorption spectra were π to π* type. A positive development in the oscillator strength was observed, i.e., it was largest from S_0_ to S_1_ in solvent compared with the gas phase. According to Table 3, the computed excitation energy for all studied compounds, except **Comp3** in solvent, was almost the same. All of the compounds exhibited two absorption peaks related to the benzothiazole part, and the charge transfer was associated with the thiophene/furan substituted moiety (Figure 5). The HOMO was entirely located on the molecules and benzothiazole, while the LUMO was on the thiophene-furan part, which was an illuminating charge transfer-type transition (CT) of the π to π* in the first bands in the λ_max_.

The lowest energy gaps were the π to π* for **Comp3** (2.8 eV), while the other benzothiazole derivatives showed larger band gaps as established from the absorption values (see Table 3). These results clearly showed that the interaction between the donor and the acceptor, either in an alternating manner or in a separate block in the molecule, played an important role in controlling the planarity and the photophysical properties [58].

### 3.3. Charge Transport Properties

Charge transports are sensitive to internal traps [59] as well as transport within p-type materials from low ionization potential levels, making the filling of deep traps less attractive. Likewise, materials of *n*-type benefit from a larger EA, where there are also fewer available traps. The hole injection from an electrode to a semiconductor HOMO is more effective when the electrode is closer, or even greater than a semiconductor IP. Similarly, for better electron injection, larger EA values would be suitable. For better stability of the device, it was certified that its charged and neutral states do not contribute to chemical reactions [60]. To preclude the thermodynamically efficient reaction (which contains oxygen and water), a neutral semiconductor is expected to require an ionization potential greater than 4.9 eV [60]. The shallow HOMO may diminish ambient O_2_ in the H_2_O existence to form OH^−^. The semiconductors having a deep HOMO can receive holes that oxidize the H_2_O in the atmosphere. Obstacles encountered during such an undesirable reaction by chance lead to overpotentials, which permits organic semiconductors to gently make redox to unveil the stabilities. Electron transport is most affected by the reaction with air, and it is important to prevent the electron polaron from degrading the material. To achieve this, the LUMO must be low to avert excited electrons from reducing the water-soluble O_2_ systems to O_2_^−^ [61] or H_2_O to OH^−^. These undesirable electrochemical processes can reduce charge transfer and set about irreversible changes within the semiconductor. Defining the exact amount of EA that needs to be skipped to prevent this redox reaction requires consideration of the maximum response power and device morphology. It has been suggested that high EA can put down oxidation reactions [62].

The values of the ionization potential (IP) and the electron affinity (EA) are among the most important factors of organic compounds in xerography and electroluminescence. These parameters were interconnected to the amount of energy required to add or remove electrons and thus were considered as molecular stability with respect to donating or receiving electrons to create an exciton. The IPs and EAs for all systems are listed in Table 4. In addition, this entailed a positive correlation between the LUMO level and EAs. We set them as the injection of an electron is affected by the HOMO and LUMO levels. The π-conjugated organic materials with an electronic charge motion were created by a hopping mechanism. The reorganization energy (λ) as an important factor influencing the rate of the hopping charge because of its structural variation from neutral to ionic states (cation and/or anion). In order to have a high degree of material mobility, the λ must be reduced. The λ is a significant parameter for the estimation of the rate of charge transfer. Previously, it was discovered that the DFT can be a reliable way to replicate the experimental data [63,64,65,66]. The internal reorganization (λ_int_) and external polarization (λ_ext_) are two components of λ [67]. Here, λ_int_ was projected for the hole (λ_hole_) and electron (λ_elec_). The λ_hole_ was estimated by Equations (1) and (2) [68]:

λ_1_ = E^+^ (B) − E^+^ (B^+^), (1)

λ_2_ = E (B^+^) − E (B) (2)

where E^+^ (B), E^+^ (B^+^), E (B^+^), and E (B) are the energies of the cation at neutral optimized geometry, neutral at the cationic optimized geometry, optimized cation, and optimized neutral geometry, respectively. Correspondingly, λ_elec_ was estimated by Equations (3) and (4):

λ_3_ = E^−^ (B) − E^−^ (B^−^), (3)

λ_4_ = E (B^−^) − E (B) (4)

where E^−^ (B), E^−^ (B^−^), and E (B^−^) are energies of the anion at neutral optimized geometry, neutral at anionic optimized geometry, and the anionic optimized geometry.

The vertical and adiabatic EA and IP were assessed by applying the following equations:

IP_a_ = E(B^+^) − E(B), (5)

EAa = E(B) − E(B^−^), (6)

IP_v_ = E^+^(B) − E(B), (7)

IP_v_ = E^+^(B) − E(B), (8)

The computed (λ_hole_ and λ_elec_) and λ_int_ of the benzothiazole derivatives at the level of B3LYP/6-31+G** are shown in Table 4. The λ_hole_ was calculated to be much smaller than the λ_elec_, with the difference in the range of 0.071 to 0.271 eV. Hitherto, it was pointed out that lower λ values can increase the charge transfer rate [65,69]. The substitution of -NO_2_ at position 5 of the furan in **Comp3** led to a decrease in the polarization of the neutral to cation state, which meant that the λ_hole_ values were lower compared with other compounds, suggesting that this compound was a good choice for the hole transfer. It may turn out that the -CH_3_ group at position 5 of furan in **Comp4** led to a decrease in the polarization of the neutral to anion, which will result in a lower λ_elec_ value in comparison with other compounds, suggesting that this compound may be a good candidate for electron transport. Here we also compared the λ_hole_ value of the benzothiazole derivatives with TPD (λ_hole_ = 0.290 eV) [70]. The λ_hole_ values of the benzothiazole derivatives were much lower than those of the best practice TPD, which suggested that the compounds we studied may be better hole transfer contenders.

### 3.4. Molecular Electrostatic Potential

**Figure 7:**
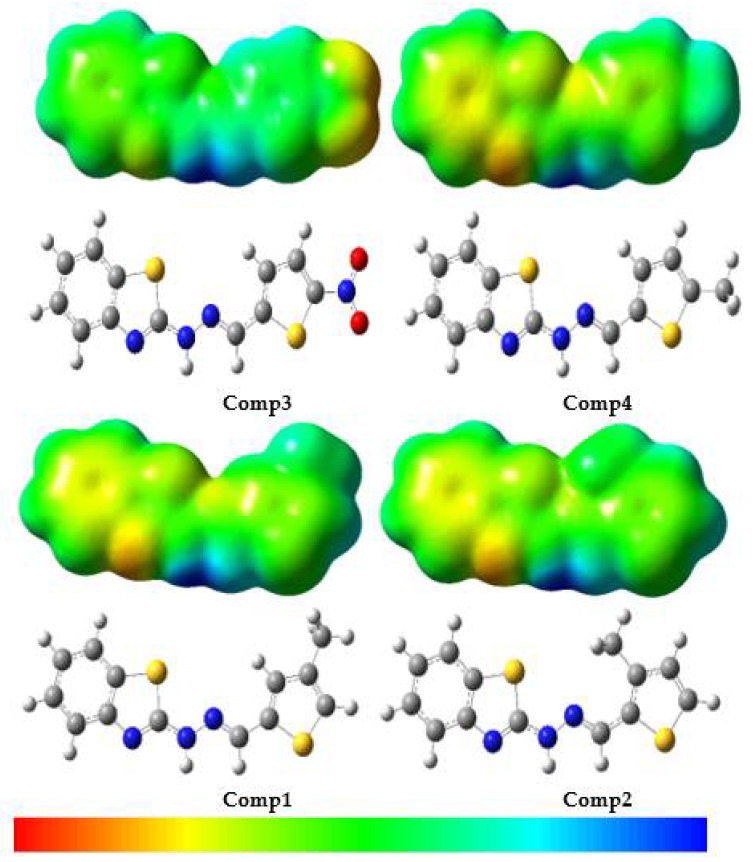
Molecular electrostatic potential surfaces views of benzothiazole derivatives.

Earlier works reported that diffraction methods would experimentally determine the molecular electrostatic potential (MEP) [62,63]. In addition, it could be tested by simulation. The MEP measures the widespread electronic distribution across the board, which is a broad aspect of understanding and predicting the reactivity of different compounds [64]. Figure 7 contains the MEP map in color. Red represents regions with high negative potential and blue indicates regions with high positive potential. The high potentials of negative/positive regions are ideal for electrophilic/nucleophilic attacks. The MEP decreases in the order blue > green > yellow > orange > red; red indicates the greatest repulsion while blue indicates the most attractive phase of the nucleophilic attack.

Figure 7 reveals that the nitrogen atom in the benzothiazole moiety, in particular, had negative potential, while -NH on the bridge with the -CH_3_ group had good positive potential. In **Comp3**, the -NO_2_ group had a negative potential. These results clearly indicated that in the event of a nucleophilic attack, the repulsion will be in potentially negative MEP areas, i.e., the benzothiazole moiety, especially the nitrogen atom and the -NO_2_ group, while the main attraction will be on the -NH and -CH_3_ groups. In addition, in the event of an electrophilic attack, the attraction may be to the benzothiazole moiety, especially the nitrogen atom and the -NO_2_ group, while the greater repulsion may be to the atoms in the -NH and the -CH_3_ groups.

### 3.5. UV–vis Experiment, Thin Film Preparation and Characterization

#### 3.5.1. UV–vis Experiment

**Figure 8:**
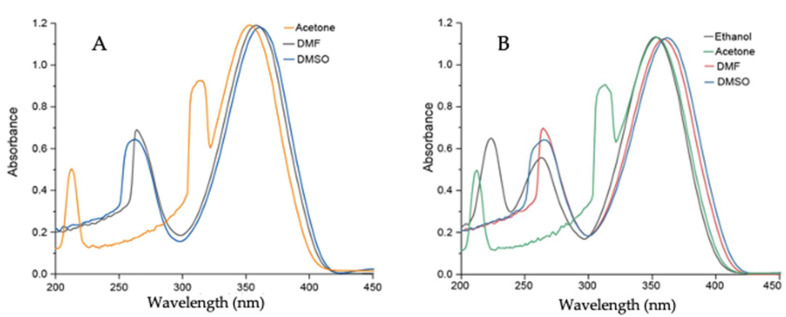
UV–vis spectrum of (**A**) **Comp1** (0.5 × 10^−5^ M) in DMF and DMSO; (**B**) **Comp2** (0.5 × 10^−5^ M) in acetone, ethanol, DMF, and DMSO.

**Figure 9:**
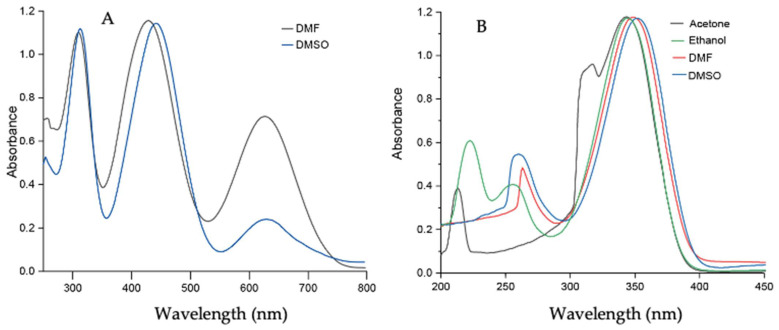
UV–vis spectrum of (**A**) **Comp3** (0.5 × 10^−5^ M) in DMF and DMSO; (**B**) **Comp4** (0.5 × 10^−5^ M) in acetone, ethanol, DMF, and DMSO.

A set of UV–vis experiments was performed in various polar solvents to gain insight into the effect of solvent polarity on **Comp1–4**. **Comp1**, **Comp2**, and **Comp4** produced similar results because of the presence of a similar D-A system as well as methyl substituents. **Comp3** produced entirely different results because of the presence of strong acceptor -NO_2_ groups, which facilitated charge transfer more effectively than **Comp1**, **Comp2**, and **Comp4**. The peaks at 350–360 nm and 250–270 nm corresponded to the n-p* and p-p* transitions for **Comp1–4**. The additional peak in **Comp3** at 430–445 nm was due to the presence of charge transfer. In addition, upon increasing the polarity of the solvent, a sequential red shift was observed, signifying the effect of solvent polarity on the D-A-based materials, see Figure 8 and Figure 9. The experimental results supported the observation made from computational data.

#### 3.5.2. Thin Film Preparation

The synthesized compounds **Comp1**, **Comp4**, **Comp2**, and **Comp3** were separately dissolved in chloroform and DMSO to obtain 5 × 10^−2^ M solutions of the compounds. The prepared solutions were individually dropped onto a precleaned FTO glass substrate. The substrate was then placed on the spin coater (Holmarc, Model no-HO-TH-05) and rotated at 1000 rpm for 30 s. This process was repeated three times to achieve a uniform film on the substrate. The spin-coated film was then annealed at 70 °C in an oven. In addition, silver contact was provided by applying silver paste on top of the film to fabricate a device in a sandwich-like fashion (FTO-**Comp**-Ag). We also used Al contact for HIE and EIE measurements of the compounds.

#### 3.5.3. Thin Film Characterization

**Figure 10:**
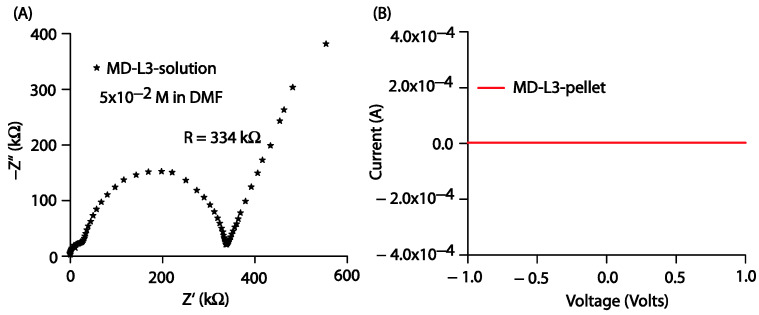
(**A**) Nyquist impedance plot for **Comp2** solution in DMF (5 × 10^−2^ M), (**B**) I-V characteristics for **Comp2** pellet.

The current-voltage (I–V) characteristics of the prepared thin films were obtained using an electrochemical workstation (model CHI608E, CH Instruments) in linear sweep voltammetry mode at a scan rate of 100 mV/s. All the prepared thin films were subjected to the applied voltage; however, in response to the applied voltage, the prepared films failed to provide any output current, as shown in Figure S17. Furthermore, to gain insight into the conductance property of the prepared compounds in bulk, we performed electrochemical impedance spectroscopic (EIS) measurements using a 3-electrode system. The prepared solution of **Comp2** in DMF was transferred into a cylindrical cell. The Nyquist impedance plot shown in Figure 10A provides the resistance of the **Comp2** solution as 334 kW. We also tried I-V measurements on the **Comp2** pellet, but there was no current in response to the applied voltage between −1 and 1 volt. We were likely unable to detect a significant current from the thin film because of its higher resistance value in bulk. However, further work related to molecular engineering to engage lone pairs with the objective of improving current response is under investigation.

## 4. Conclusions

The electronic, charge transfer, and optical properties of benzothiazole compounds were tuned. The absorption wavelengths were tested at the TD-B3LYP/6-31+G** level. Modification of chemical structures can greatly balance and improve electronic and charge transfer properties. In fact, the combination of benzothiazole with the furan unit and the -NO_2_ group resulted in a better separation of the absorption in the solar spectrum. The absorption maxima of this compound were red-shifted in the UV–vis region, expecting the blue region in the range of 481 nm in ethanol at the TD-B3LYP/6-31+G** level, which makes this material also suitable for efficient OFETs. The introduction of the -NO_2_ group into the furan at position 5 in **Comp3** led to a red shift in the absorption spectra compared with other molecules. **Comp1**, **Comp2**, and **Comp4** produced similar results because of the presence of a similar D-A system as well as the methyl substituents, while **Comp3** produced entirely different results because of the presence of strong acceptor -NO2 groups, which facilitated charge transfer more effectively than **Comp1**, **Comp2**, and **Comp4**. Values of hole reorganization energies smaller than those of electrons would improve intrinsic hole mobility. The smaller electron reorganization energy value for **Comp4** compared with other counterparts might lead to its better electron charge transfer ability. The insignificant current from the thin film was due to its higher resistance value in bulk. The reason we obtained insignificant hole/electron injection energy for the synthesized compounds was likely due to the presence of the free –NH group in the proposed molecules, resulting in the lowering of hole/electron mobility.

## Figures and Tables

**Table 1:** The HOMO/LUMO energies (E_HOMO_/E_LUMO_) and energy gaps (E_gap_) of benzothiazoles at the B3LYP/6-31+G** and TD-B3LYP/6-31+G** levels for S_0_ and S_1_ (in eV), respectively.

Compounds	E_HOMO_	E_LUMO_	E_gap_	E_HOMO_	E_LUMO_	E_gap_
	Ground State			Excited State		
**Comp1**	−5.59	−1.95	3.64	−4.77	−2.61	2.16
**Comp2**	−5.58	−1.88	3.70	−4.76	−2.55	2.21
**Comp3**	−6.18	−3.35	2.83	−5.13	−4.01	1.12
**Comp4**	−5.52	−1.92	3.60	−4.73	−2.56	2.17

**Table 2:** The electron/hole injection energy (EIE/HIE) barriers in eV from benzothiazoles to silver (Ag)/aluminum (Al) electrodes calculated at the B3LYP/6-31+G** level.

Compounds	HIE (Ag)	EIE (Ag)	HIE (Al)	EIE (Al)
**Comp1**	0.85	2.79	1.51	2.13
**Comp2**	0.84	2.86	1.50	2.20
**Comp3**	1.44	1.39	2.10	0.73
**Comp4**	0.78	1.82	1.44	2.16

**Table 3:** The absorption wavelengths (λ_max_, nm), oscillator strengths (*f*), percent contribution, and key transitions in the gas phase as well as in solvents (ethanol, acetone, DMF, and DMSO) of benzothiazole derivatives at the TD-B3LYP/6-31+G** level.

Comp	λ_max_	*f*	Tran	%Con	λ_max_	*f*	Tran	%Con
	Gas phase				In Ethanol			
**Comp1**	358	0.8839	H→L	70%	363	1.1034	H→L	70%
	281	0.2282	H→L + 1	30%	281	0.1313	H→L + 1	29%
**Comp2**	357	0.8301	H→L	70%	359	1.0638	H→L	70%
	278	0.2280	H→L + 1	35%	277	0.1452	H→L + 1	39%
**Comp3**	451	0.6867	H→L	70%	481	0.8210	H→L	70%
	323	0.4853	H→L + 1	27%	335	0.4279	H→L + 1	23%
**Comp4**	362	0.9571	H→L	70%	367	1.1864	H→L	70%
	284	0.2341	H→L + 1	29%	283	0.1312	H→L + 1	31%
**Comp**	λ_max_	*f*	Tran	%Con	λ_max_	*f*	Tran	%Con
	In acetone				In DMF			
**Comp1**	363	1.1018	H→L	70%	364	1.1213	H→L	70%
	281	0.1323	H→L + 1	29%	281	0.1280	H→L + 1	29%
**Comp2**	359	1.0620	H→L	70%	360	1.0840	H→L	70%
	277	0.1461	H→L + 1	39%	277	0.1429	H→L + 1	39%
**Comp3**	481	0.8202	H→L	70%	484	0.8388	H→L	70%
	335	0.4287	H→L + 1	23%	336	0.4224	H→L + 1	23%
**Comp4**	367	1.1847	H→L	70%	368	1.2041	H→L	70%
	283	0.1322	H→L + 1	31%	283	0.1280	H→L + 1	31%
**Comp**	λ_max_	*f*	Tran	%Con				
	In DMSO							
**Comp1**	364	1.1189	H→L	70%				
	281	0.1278	H→L + 1	29%				
**Comp2**	360	1.0813	H→L	70%				
	277	0.1425	H→L + 1	39%				
**Comp3**	484	0.8357	H→L	70%				
	336	0.4228	H→L + 1	23%				
**Comp4**	368	1.2018	H→L	70%				
	283	0.1278	H→L + 1	31%				

**Table 4:** Adiabatic/vertical ionization potential (IP_a_/IP_v_), adiabatic/vertical electron affinity (EA_a_/EA_v_), and electron/hole reorganization energies ($\lambda_{\mathrm{elec}}/\lambda_{\mathrm{hole}}$) in eV of benzothiazoles at the B3LYP/6-31+G** level.

Compounds	IP_a_	EA_a_	IP_v_	EA_v_	$\lambda_{hole}$	$\lambda_{elec}$	Δλ
**Comp1**	7.07	0.41	6.96	0.52	0.235	0.327	0.092
**Comp2**	7.05	0.36	6.95	0.46	0.233	0.340	0.107
**Comp3**	7.64	2.09	7.52	1.85	0.216	0.487	0.271
**Comp4**	6.98	0.39	6.88	0.51	0.249	0.320	0.071

## Data Availability

Not applicable.

## Supplementary Materials

The following supporting information can be downloaded at: https://www.mdpi.com/article/10.3390/molecules27248672/s1, Figures S1–S4: Characteristics of Comp1; Figures S5–S8: Characteristics of Comp2; Figures S9–S12: Characteristics of Comp3; Figures S13–S16: Characteristics of Comp4; Figure S17: I-V characteristics of (A) Comp1-film, (B) Comp2-film, (C) Comp3-film and (D) Comp4-film; Table S1: λ_max_ for all the four compounds in acetone, ethanol, DMF and DMSO.
