# Supplementary material for: Investigation of the Effect of Substituents on Electronic and Charge Transport Properties of Benzothiazole Derivatives
Source: Molecules. 2022 Dec 8;27(24):8672. doi: 10.3390/molecules27248672 (PMC9781107; doi:10.3390/molecules27248672)
Supplement: Supplementary file 1 [file molecules-27-08672-s001.zip › molecules-2050812-supplementary.pdf]

Supporting information

Investigation of the Effect of Substituents on Electronic and Charge Transport Properties of Benzothiazole Derivatives

Ahmad Irfan [1,2,*], Abul Kalam [1], Abdullah G. Al-Sehemi [1] and Mrigendra Dubey [3]

[1] Department of Chemistry, College of Science, King Khalid University, P.O. Box 9004, Abha 61413, Saudi Arabia
[2] Research Center for Advanced Materials Science, College of Science, King Khalid University, P.O. Box 9004, Abha 61413, Saudi Arabia
[3] Soft Materials Research Laboratory, Discipline of Metallurgy Engineering and Materials Science, Indian Institute of Technology Indore, Indore 453552, India
* Correspondence: irfaahmad@gmail.com; Tel.: +00-9661-7241-9481; Fax: +00-9661-7241-8426

1. Synthesis of Comp1:

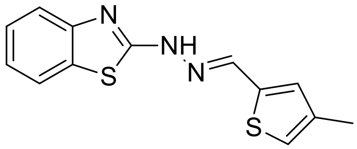

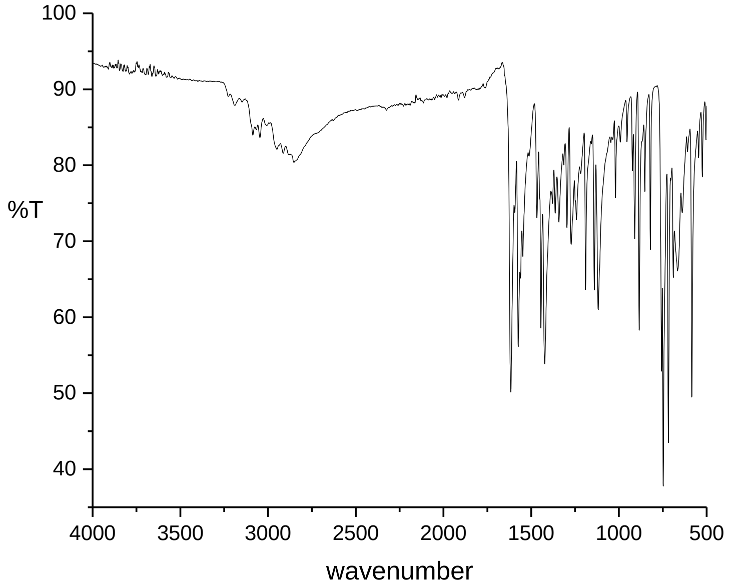

Figure S1. FTIR spectrum of ligand of **Comp1**.

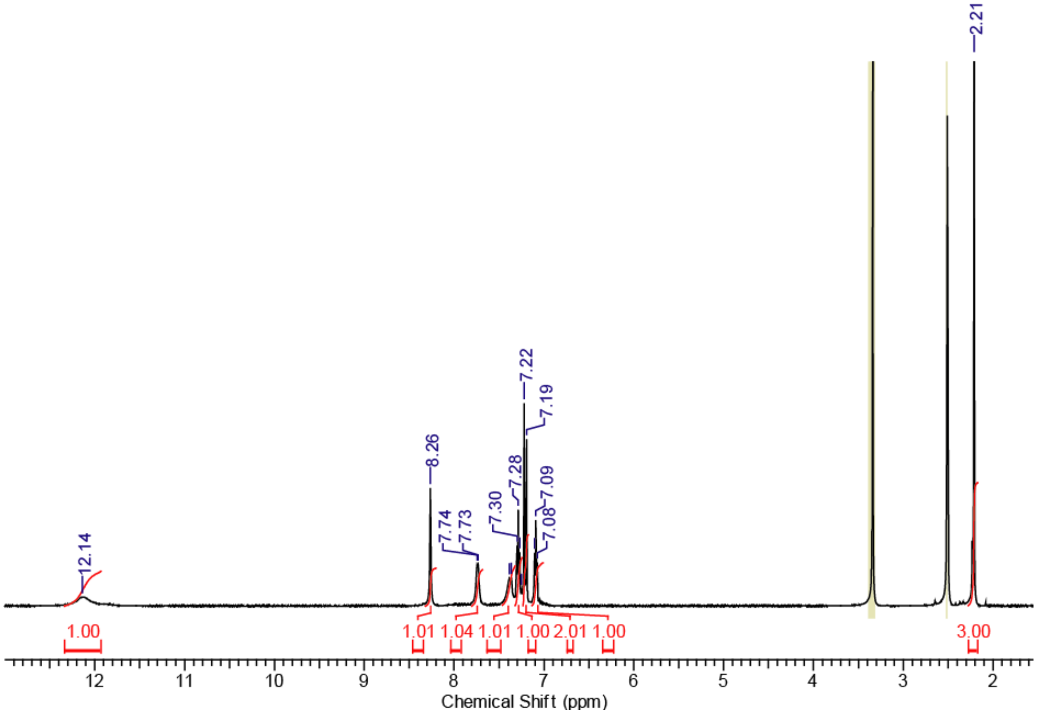

Figure S2. ^{1}H NMR spectrum of **Comp1**.

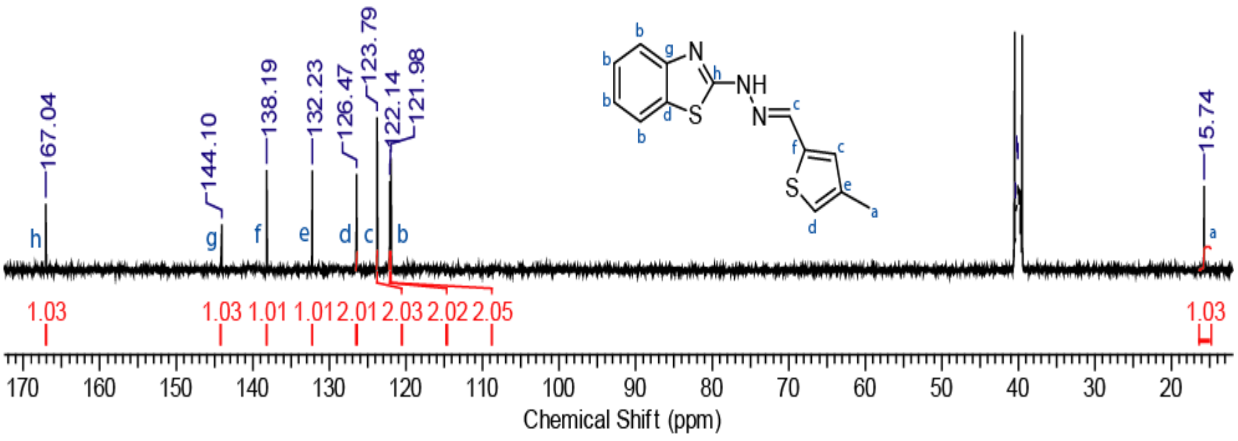

Figure S3. ^{13}C NMR spectra of **Comp1**.

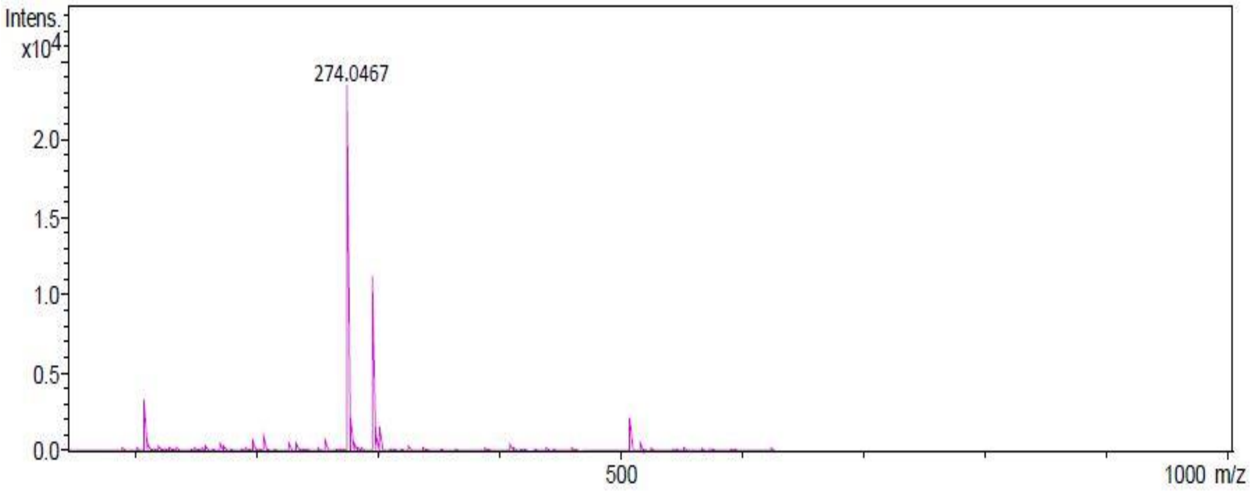

Figure S4. HR-MS spectra of **Comp1**.

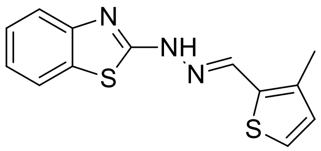

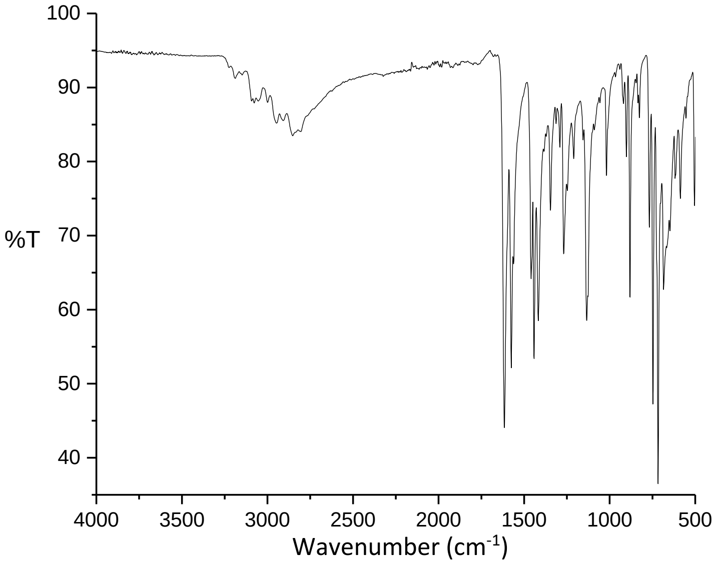

Figure S5. FTIR spectrum of ligand of **Comp2**.

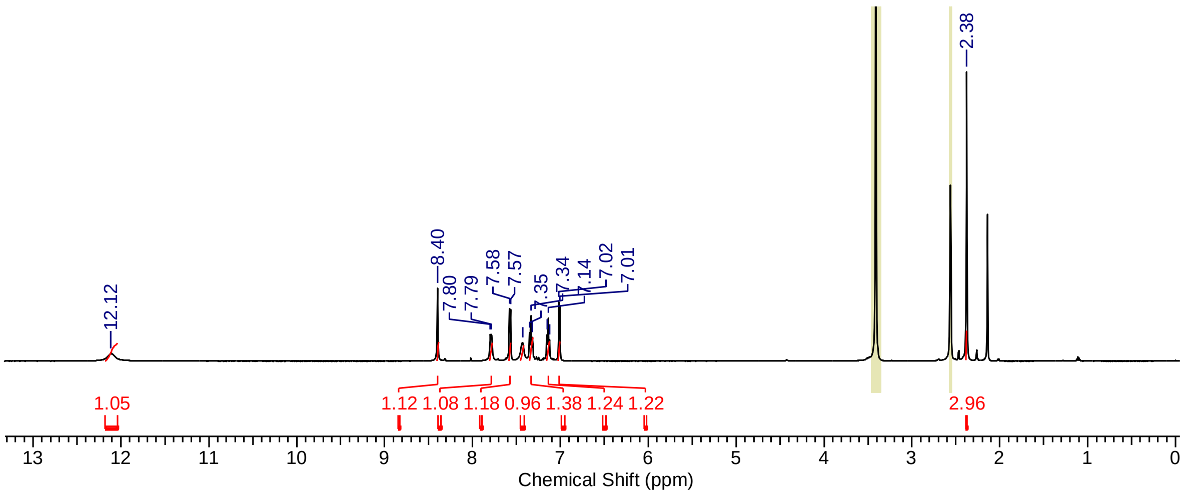

Figure S6. ^{1}H NMR spectrum of **Comp2**.

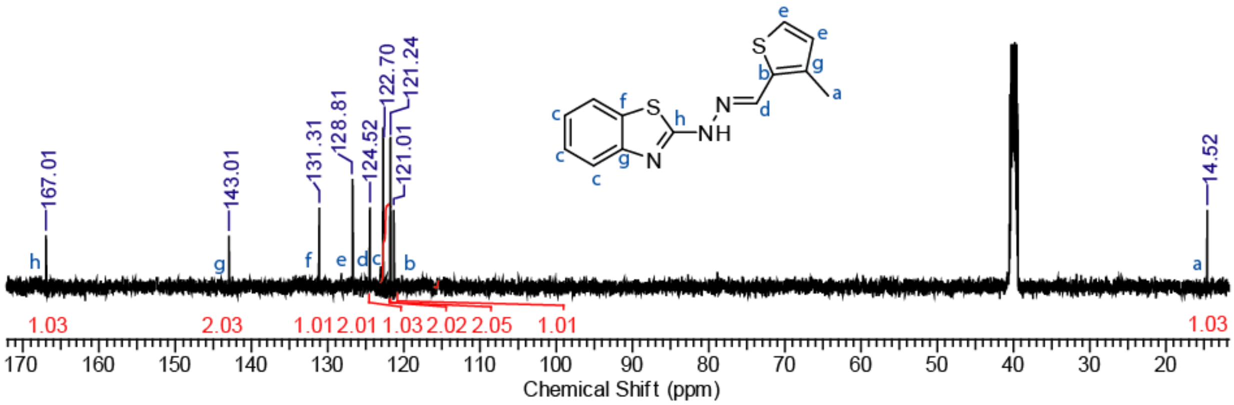

Figure S7. ^{13}C NMR spectra of **Comp2**.

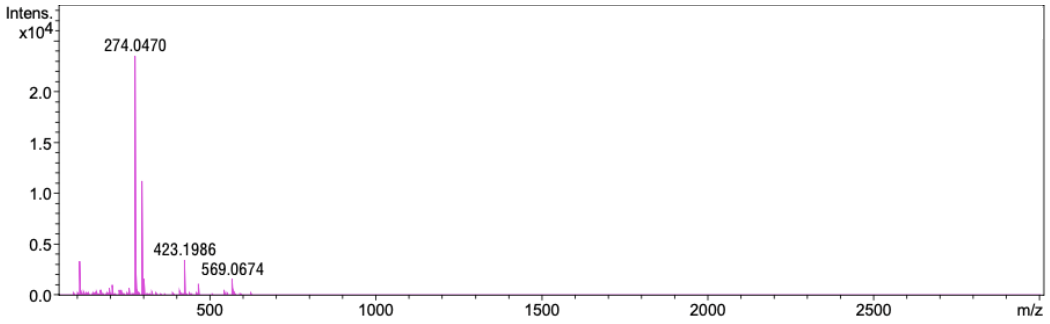

Figure S8. HR-MS spectra of **Comp2**.

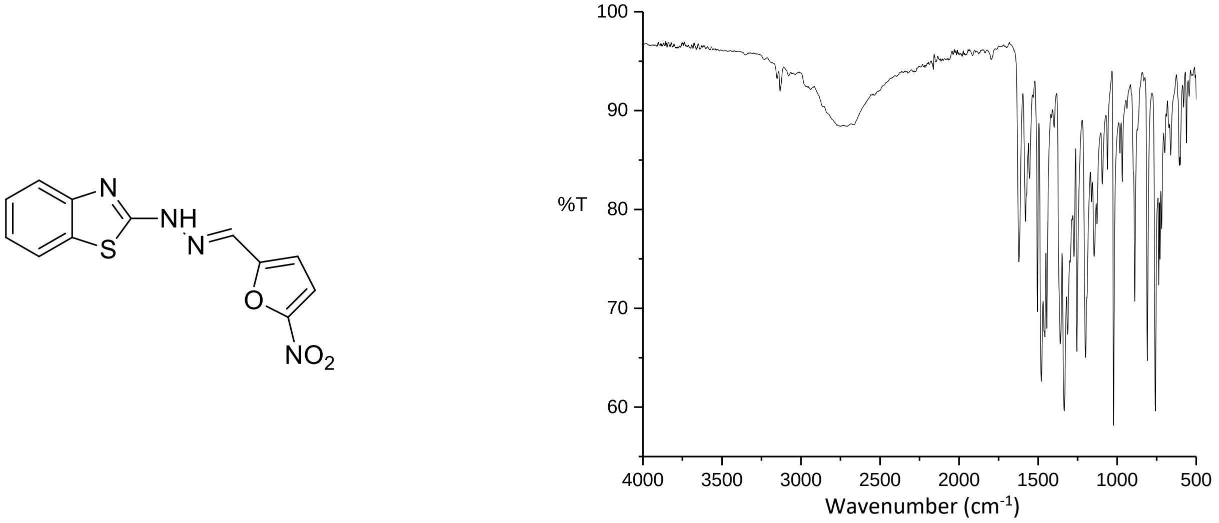

Figure S9. FTIR spectrum of **Comp3**.

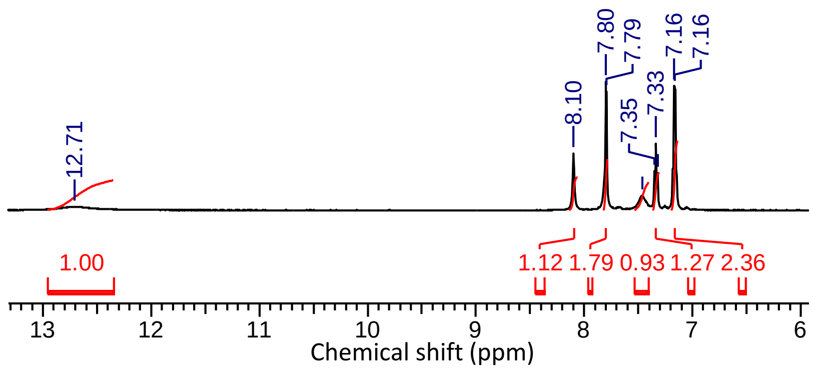

Figure S10. ^{1}H NMR spectrum of **Comp3**.

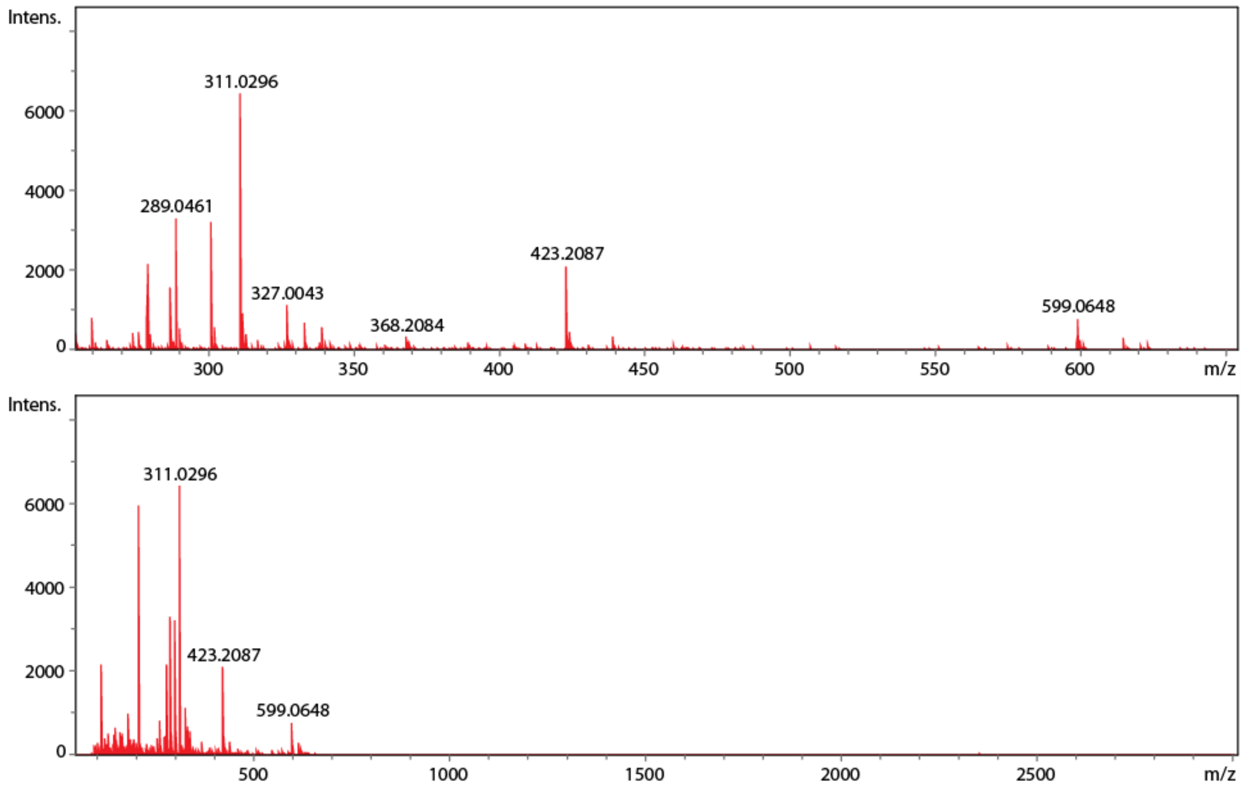

Figure S11. (Lower) Full HR-MS spectrum and (upper) selected HR-MS spectrum of **Comp3**.

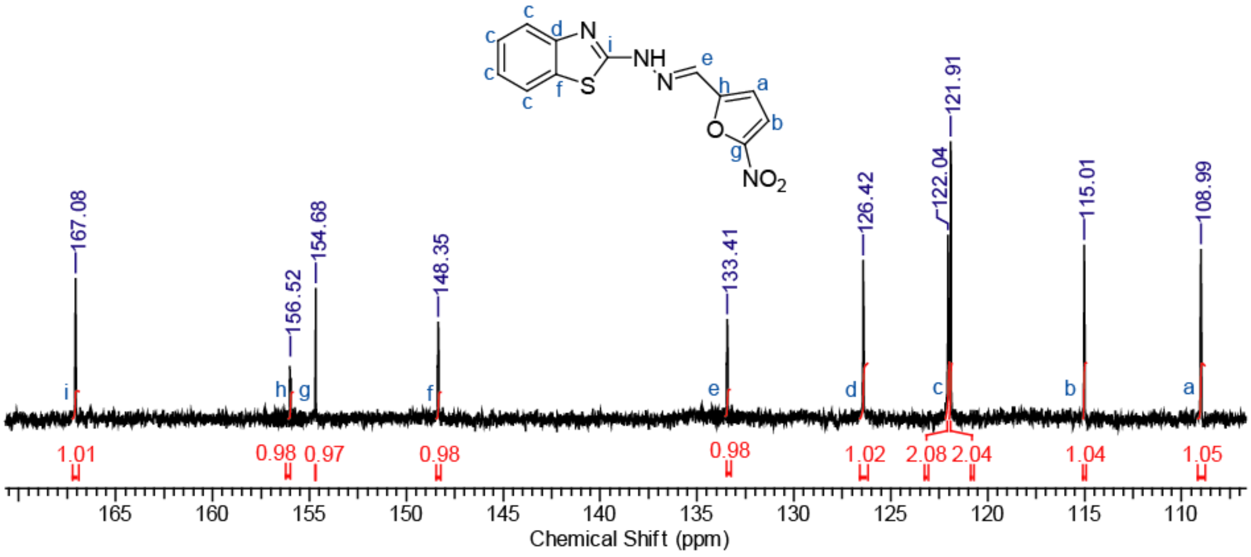

Figure S12. ^{13}C NMR spectra of **Comp3.**

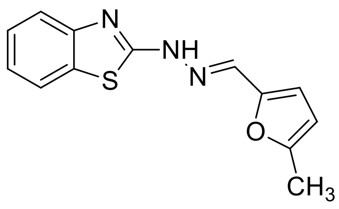

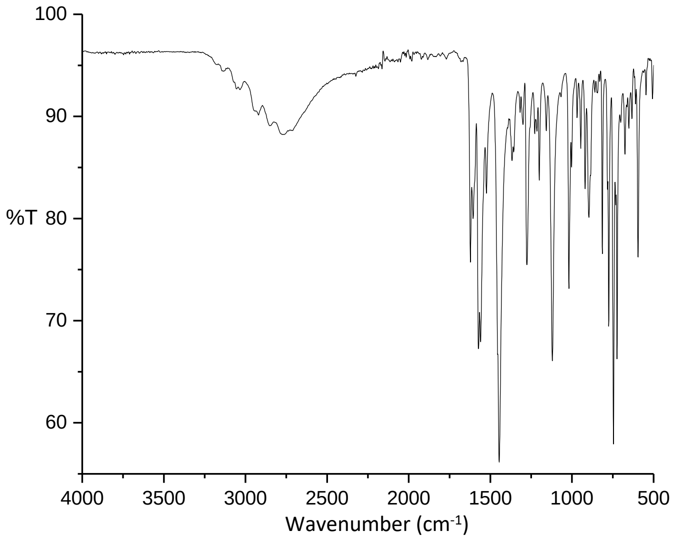

Figure S13. FTIR spectrum of **Comp4**.

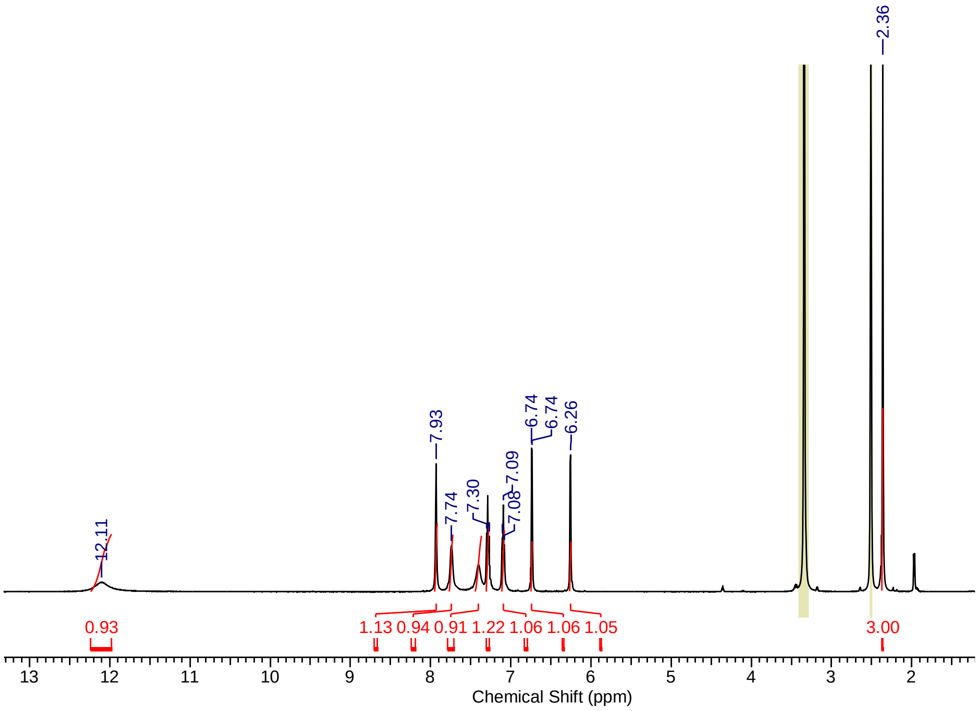

Figure S14. ^{1}H NMR spectrum of **Comp4**.

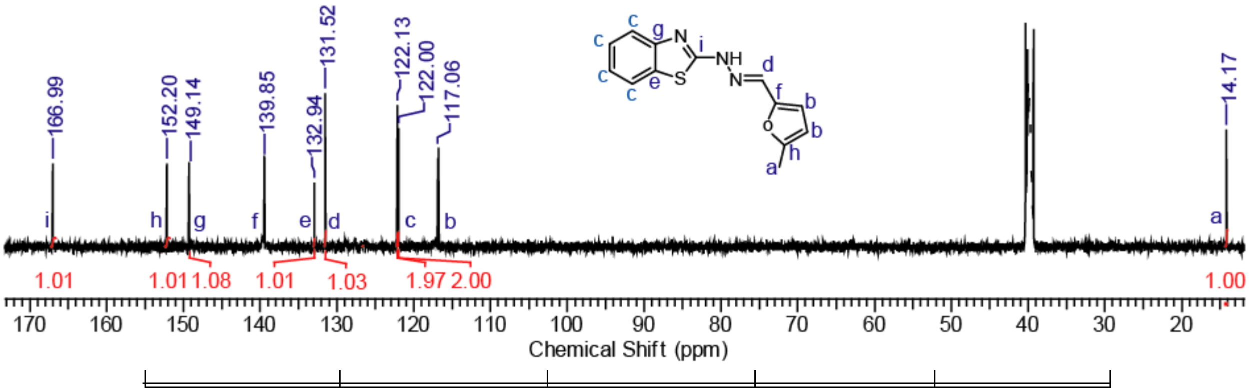

Figure S15. ^{13}C NMR spectra of **Comp4**.

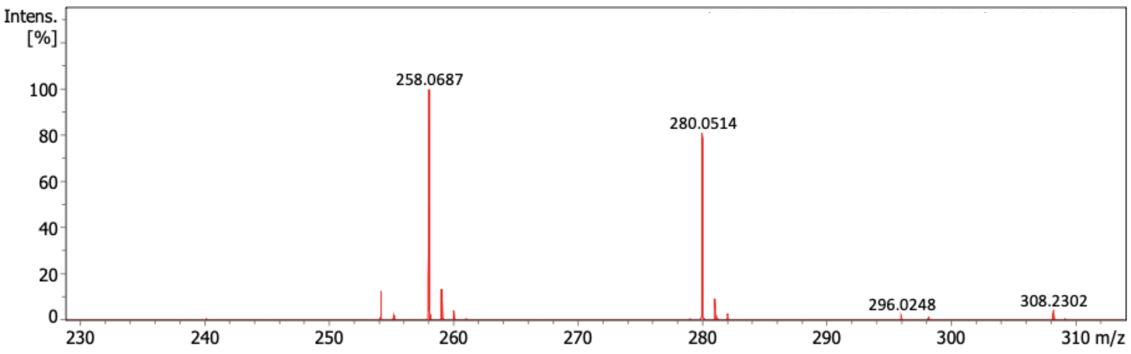

Figure S16. HR-MS spectrum of **Comp4**.

Table S1. λ_{max} for all the four compounds in acetone, ethanol, DMF and DMSO.

**Compounds**	Acetone λ_{max} (nm)	Ethanol λ_{max} (nm)	DMF λ_{max} (nm)	DMSO λ_{max} (nm)
**Comp1**	353	Insoluble	360	362
**Comp2**	353	352	358	361
**Comp3**	Insoluble	Insoluble	430	445
**Comp4**	343	344	350	353

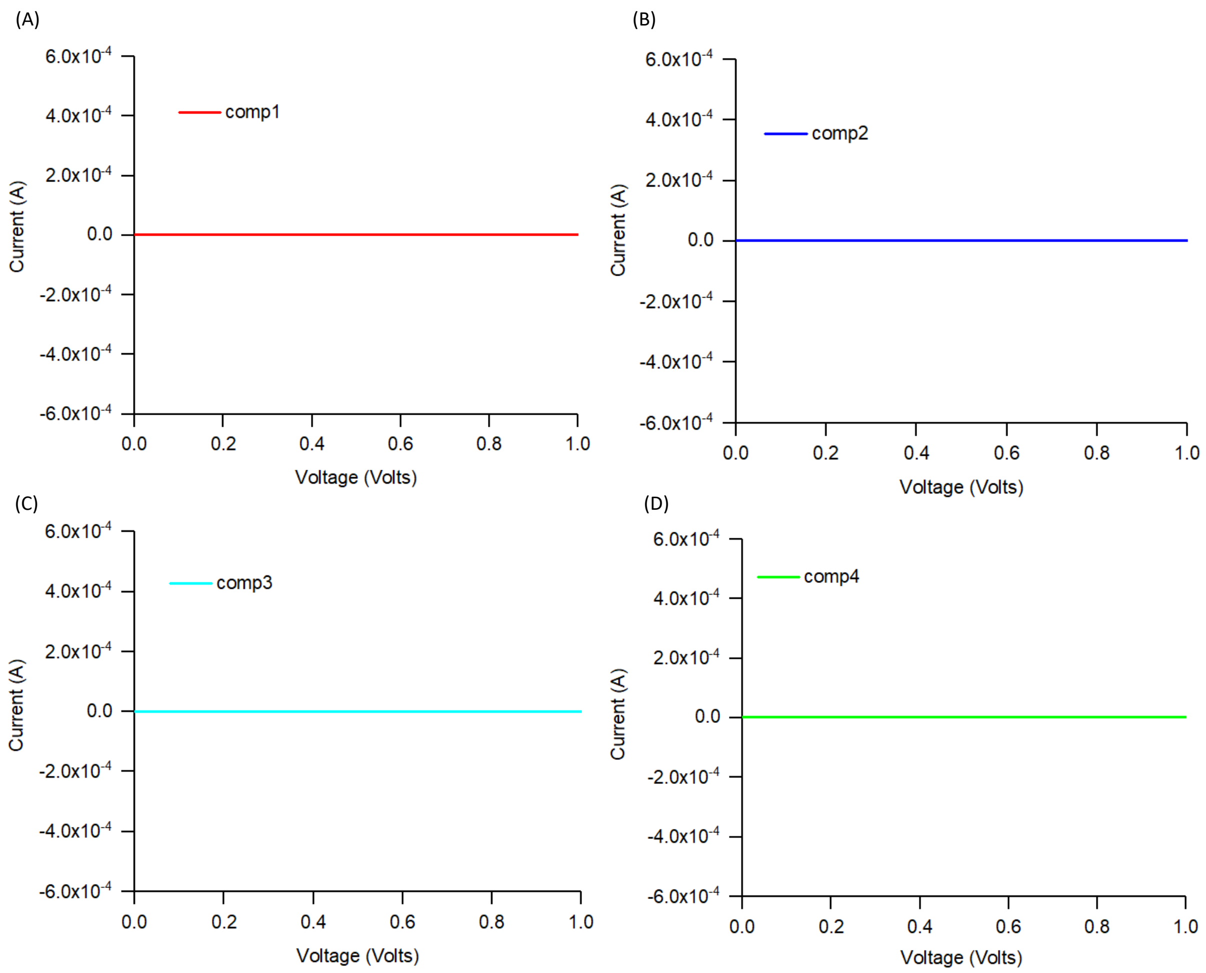

Figure S17. I-V characteristics of (A) **Comp1**-film, (B) **Comp2**-film, (C) **Comp3**-film and (D) **Comp4**-film.
